# Link between children's oppositional behaviors and parental quality of life post‐ASD diagnosis: Mediating role of parental stress and coping strategies

**DOI:** 10.1002/jcv2.12303

**Published:** 2025-01-09

**Authors:** Cécile Rattaz, Andrew Pickles, Christelle Vernhet, Cécile Michelon, Marie‐Christine Picot, Amaria Baghdadli

**Affiliations:** ^1^ Centre de Ressources Autisme Languedoc‐Roussillon et Centre d'excellence sur l’Autisme et les Troubles du Neuro‐développement (CeAND) CHU Montpellier Montpellier France; ^2^ Université Paris‐Saclay UVSQ Inserm CESP Villejuif France; ^3^ Department of Biostatistics and Health Informatics Institute of Psychiatry Psychology & Neuroscience King's College London London UK; ^4^ Clinical Research and Epidemiology Unit Department of Medical Information University Hospital CHU Montpellier Montpellier France; ^5^ Faculté de Médecine Université de Montpellier Montpellier France

**Keywords:** autism spectrum disorder, coping, oppositional behaviors, parental QoL, parental stress

## Abstract

**Background:**

Oppositional behaviors are often an important issue for parents who have a child with autism spectrum disorder (ASD), and have been shown to be related to their Quality of Life (QoL). The present study examines the possible mediation and moderation effects of parenting stress and coping in the relationship between oppositional behaviors in the child with ASD and parental QoL.

**Method:**

Parental stress, coping strategies, parental QoL and children's challenging behaviors (CBCL oppositional problem domain) were studied through parent‐report (335 mothers and 230 fathers) over two occasions in a cohort of 485 children and adolescents with ASD (398 boys and 87 girls) with a mean age of 6.14 years (SD = 3.46) at baseline. Mediation and moderation effects were examined using structural equation modeling.

**Results:**

Strong associations between child behaviors, parental stress and parental QoL were evidenced. Parental stress mediated the relationship between opposition in the child and parental QoL at diagnosis, but this effect was much smaller 3 years after diagnosis. Surprisingly, problem solving and social support, two commonly recommended coping strategies, had little or no effect on reducing impact on QoL 3 years later.

**Conclusions:**

Longitudinal analysis allowed us to estimate the causal pathway between child oppositional behaviors, parental stress and impact on parental QoL. Our findings emphasize the crucial role of parental stress, which can mediate the impact of the children's oppositional behaviors on parental QoL. They argue for the need to develop specific interventions for parents focusing on parental stress and child's behavior management.

## Graphical abstract

This paper examines the mediating effect of parental stress and coping strategies on the relationship between challenging behaviors and parental quality of life at diagnosis and 3 years later. We found that parental stress acts as a buffer between opposition in the child and parental QoL at ASD diagnosis, but that this effect was much smaller 3 years after.

**Key points**

**What's known**

- Oppositional behaviors in children with autism spectrum disorder (ASD) impact parental Quality of Life (QoL).

**What's new**

- Parental stress acts as a buffer between opposition in the child and parental QoL at ASD diagnosis, but this effect is much smaller 3 years after. Parental coping strategies at diagnosis are not sufficient to lead to a decrease in child's challenging behaviors 3 years later.

**What's relevant**

- Early interventions should focus on comorbid disorders, which are a red flag concerning a decreased parental QoL during the diagnosis period. In the future, more longitudinal studies are needed to better understand the role of stress and coping strategies across time.

## INTRODUCTION

Parenting a child with ASD can be highly stressful and challenging for many parents, who are at greater risk for decreased QoL compared to families of typically developing children (see Vasilopoulou and Nisbet (2016) for a review). The concept of QoL is defined as “individuals' perception of their position in life in the context of the culture and value systems in which they live and in relation to their goals, expectations, standards and concerns” (World Health Organization, 1993). Gender differences were evidenced in the literature, with a lower level of satisfaction regarding QoL in mothers than in fathers (Dabrowska & Pisula, 2010; Vernhet et al., 2022).

The impact of ASD on parental QoL was shown to be related to child characteristics, notably comorbid disorders which are highly prevalent in this population (Anixt et al., 2020; Baghdadli et al., 2014; Martin et al., 2024; Matson & Nebel‐Schwalm, 2007; Rattaz et al., 2018; Vitale et al., 2023). Comorbid psychopathology in children with ASD include anxiety, depression, conduct and oppositional defiant disorders and attention‐deficit/hyperactivity disorder (Anixt et al., 2020; Leader et al., 2021). Among challenging behaviors, externalizing behaviors (e.g., conduct problems such as opposition, aggressive, destructive behaviors) have been found to significantly impact family functioning and to contribute to higher level of parental stress, lower parental QoL and greater likelihood of depressive symptoms (Adams et al., 2018; Allik et al., 2006; Estes et al., 2009; Lanyi et al., 2022; McStay et al., 2014; Vitale et al., 2023). In a previous study, we found that mothers' QoL was negatively associated with their child's internalizing (e.g., withdrawn, anxious, or depressed) disorders, whereas fathers' QoL was negatively associated with their child's externalizing disorders (Vernhet et al., 2022).

Parents of children with ASD need to develop ongoing effective responses to deal with these behaviors and several studies focused on the way parents adjust their way of reacting to maladaptive behaviors in their child with ASD over time (Dieleman et al., 2017). One of the most common response is an increase in parental stress, that can be defined as “an aversive psychological reaction to the demands of being a parent that stems from a complex combination related to the child, the parent and the child–parent interactions” (Abidin, 1995). When stress occurs, the individual develops coping strategies, which are defined as “the cognitive and behavioral efforts that are constantly changing to master, reduce or tolerate a specific stressor appraised as exceeding one's available resources and abilities” (Lazarus & Folkman, 1984). The most commonly described coping strategies are the problem‐focused strategy (which consists of acting on the problem), the emotion‐focused strategy (which aims to manage the emotional distress associated with the problem) and the seeking social support strategy (Greenglass, 1993; Lazarus & Folkman, 1984). In the literature, active problem‐focused strategies and social support were associated with parental well‐being whereas poor coping strategies (e.g., behavioral disengagement, escape avoidance, emotion‐oriented) were negatively associated with QoL (Alostaz et al., 2022; Benson, 2010; Carona et al., 2014; Dabrowska & Pisula, 2010; Dardas & Ahmad, 2015b; Fairfax et al., 2019; Hastings et al., 2005).

Recently, some studies focused on the variables that might mediate or moderate the relationship between the stressor (for example ASD symptoms) and the outcome variable (parental QoL). A mediator is a third variable that lies on the causal pathway between the stressor and the outcome variable, whereas a moderator modifies the influence of the stressor on the outcome variable (Fairfax et al., 2019; Lindley & Walker, 1993). Carona et al. (2014) found that behavioral disengagement, an avoidant emotion‐focused coping strategy, mediated the relationship between caregiving burden and parents' QoL. Lyons et al. (2010) described that parental coping strategies moderated the impact of autism symptoms on parental stress (Lyons et al., 2010). Increasing social support weakened the negative effect of parenting stress on life satisfaction (Lu et al., 2018), whereas “accepting responsibility” mediated the relationship between stress and QoL and “seeking social support” and “escape avoidance” moderated the relationship between stress and QoL (Dardas & Ahmad, 2015a). Those results are promising, however there is no studies to our knowledge that focused on the variables that might mediate or moderate the association between co‐occurring problem behaviors and parental QoL. More research is needed to better understand how stress and coping might buffer associations between comorbid behaviors and QoL, and the possible differences between mothers and fathers. Moreover, all these studies were based on cross‐sectional association, known to be a weak basis for claims of causal mediation and moderation. Longitudinal studies are needed in order to be able to make causally interpretable conclusions about the mediating and moderating effects of stress and coping strategies over time.

In this article, we used data from the ELENA cohort of children with ASD and their parents to examine whether mothers' and fathers' stress and coping strategies might mediate or moderate the associations between challenging behaviors and parental QoL both cross‐sectionally, at the time of diagnosis, and 3 years later, controlling for their pre‐existing association. We focused on oppositional defiant behaviors, which are a crucial issue for parents and were shown to be associated with parental QoL (Adams et al., 2018; Allik et al., 2006; Estes et al., 2009; Lanyi et al., 2022; McStay et al., 2014; Vernhet et al., 2022).

## MATERIALS AND METHODS

### Participants

**FIGURE 1:**
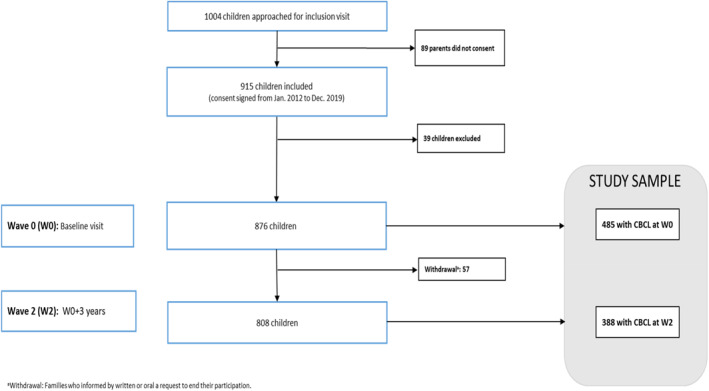
Flow chart of the ELENA cohort.

This cross‐sectional study used data from the prospective, multicenter French cohort ‐ ELENA ‐ of children and adolescents with a confirmed diagnosis of ASD (Baghdadli et al., 2019; ClinicalTrials.gov NCT02625116). Among the 1004 eligible children, 89 parents did not consent and for 39 children data about diagnosis tools were missing (see Figure 1). In total, 876 children were recruited by December 31, 2019, from 13 French autism resources centers specialized in the assessment and diagnosis of ASD and other neurodevelopmental disorders and respectful of best practices guidelines (Baghdadli et al., 2019). Inclusion criteria for the ELENA cohort were to be aged 2–16 years and diagnosed with ASD according to DSM‐5 criteria. Children were diagnosed by a multidisciplinary team according to a standardized process, including the Autism Diagnostic Interview‐Revised, the Autism Diagnostic Observation Schedule II (ADOS‐II), the Vineland Adaptive Behavior Scale‐II (VABS II), and psychometric tests to assess IQ (Wechsler, K‐ABC II, PEP, or Brunet‐Lézine scales, depending on the child's level). Parental consent was collected and the current study was approved by the Internal Review Board of University of Montpellier. Ethical approval was obtained through the Marseille Mediterranean Ethics Committee (ID RCB: 2014‐A01423‐44). There were no exclusion criteria for the ELENA cohort other than parental refusal. As part of the follow‐up (i.e., at diagnosis, 3 years after, and 6 years after), data were collected through clinical examination and online self‐completed questionnaires on the child's behavior, the socio‐demographic and psychosocial measures in a standardized manner.

In this article, we used a subset of participants from the ELENA cohort, consisting of children or adolescents whose parents completed the CBCL at baseline (see Figure 1).

### Measures

*Parenting Stress Index–Fourth Edition ‐ Short Form* (PSI‐4‐SF) consists of 36 statements, and parents respond to each statement using a five‐point scale to indicate the degree to which that item has been disturbing to them in the past week. This instrument yields scores for several factors in addition to a Total Stress score, which was used in this study. Parents who obtain a Total Stress score above a raw score of 90 are considered to experiencing clinically significant parenting stress. Internal consistency reliability for the composite Total Stress is reported by the author to be 0.91. Stability of the instrument was assessed by test‐retest after a 6‐month interval and yielded an alpha of 0.84 for the Total Stress. The French version (Bigras et al., 1996) validated in a French‐speaking population (Toucheque et al., 2016) was used.

*WCC‐R (Ways of Coping Checklist)* is a scale, inspired by Lazarus and Folkman's transactional model of stress created by Vitaliano et al. (1985), that was translated and validated in French by Cousson et al. (1996). It is a self‐completed questionnaire composed of 27 items on a four‐point Likert scale. The scores obtained from the responses to the items are analyzed through three dimensions: problem‐focused coping, emotion‐focused coping, and social‐support seeking coping. The Cronbach's alpha coefficients were 0.76, 0.73, and 0.76 for each dimension, respectively.

*Parental ‐ Developmental Disorder–Quality of Life* (Par‐DD‐QoL) was used to assess the Impact of ASD on parental QoL (IoQoL) on the following dimensions: Emotional, Daily Disturbance and Global QoL (Baghdadli et al., 2014). Par‐DD‐QoL contains 17 questions, each rated by parents on a 5 ‐ point Likert scale. The questionnaire begins with the sentence: “because of your child's disorders, do you feel….”. The first 15 questions concern the intensity of the difficulties encountered by parents, the 16th questions their frequency and the last one the global parental QoL. There are two sub‐scores, Emotional score (ES as the sum of Q1–Q6, Q13 and Q14) and Daily Disturbances score (DDS as the sum of Q7–Q11, Q15 and Q16), and a Global Score (sum of the previous 2 scores). Good internal consistency reliability was observed for the 2 subscales. Cronbach's alpha coefficient was greater than 0.82 for each dimension. As the number of items is not the equivalent in each domain, the scores were linearly transformed in a range from 0 to 100, 0 being the best and 100 the worst, assuming equal weights on each domain.

*Child Behavior Checklist (CBCL)* is a self‐rated parental questionnaire used to assess a wide range of emotional and behavioral disorders in children aged from 1.5 to 5 years (pre‐school) and 6–18 years (school‐age) (Achenbach & Edelbrock, 1983). It consists of six DSM‐oriented scales: affective problems, anxiety problems, somatic problems, attention deficit, oppositional problems, and conduct problems. The analysis focused on the oppositional scale, as oppositional behaviors were shown to be mainly related to parental stress and QoL (see Introduction). Children in the clinical or borderline clinical range are compared to children in the normal range. Raw scores are transformed into T‐scores based on published norms, with T‐scores ≥65 considered clinically significant. Internal consistency ranged from 0.72 to 0.91, the interrater reliability from 0.63 to 0.88, and the test‐reliability was 0.90.

### Statistical analysis

**FIGURE 2:**
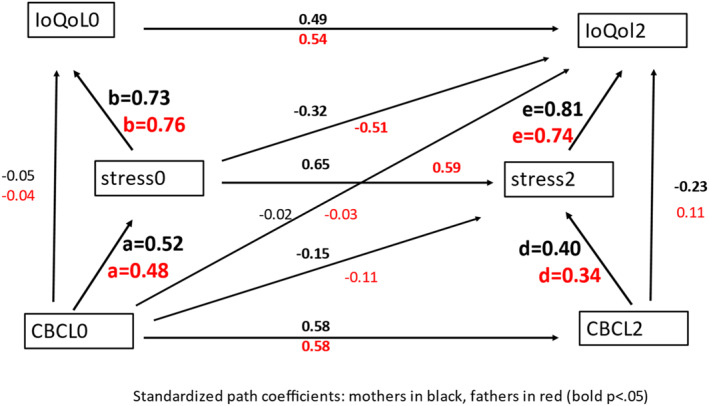
Longitudinal Mediation Model: Standardized path coefficients (mothers black, fathers red), bold significant at *p* < 0.05, main mediation path coefficients shown in larger font.

The cross‐lagged mediation model shown in Figure 2 examines the extent to which stress mediates the effects of child oppositional behavior on parental QoL. It examines this mediation pathway in the cross‐sectional data of the baseline assessment (a times b), and then also at the third assessment (d times e) while adjusting for the baseline values. These latter estimates can therefore be considered as more causally interpretable, since covarying by baseline, they more closely relate to change in predictors and associated change in outcomes. The model is estimated in Stata 17 SEM using full‐information maximum likelihood with participants requiring baseline age, sex and behavior score for inclusion. Extended models with interaction effects and two‐group models to capture possible moderation were fitted, and multiple mediator models as shown in Figure S1. SEM yields similar direct and indirect effect estimates as other causal inference methods that assume linearity in this setting (De Stavola et al., 2015). Following an analysis of factors associated with sample inclusion and attrition, inverse probability weights were calculated from a logistic regression that included all significant predictors, and alpha‐trimmed to 2.5 SD, and the analysis repeated using these weights to assess the sensitivity of the results to sample attrition. All reported significance levels and 95% confidence intervals are nominal with no correction for multiple testing. The confidence interval for the indirect effects involving the product of coefficients (the estimate of the path a coefficient multiplied by the estimate of the path b coefficient in Figure 2) were estimated using bootstrap with 500 replicates. The presence of floor‐effects in CBCL T‐scores makes it preferable to analyze CBCL raw scores in longitudinal models such as this. To account for age, sex and CBCL version (Pre‐school and School‐age) on CBCL raw scores, we replaced them by their residuals from a regression that included as predictors age, sex and CBCL version, and the interaction of age and sex, and age and version. No significant heteroscedasticity by version, age or sex was found.

## RESULTS

### The sample

Figure 1 displays the participant flow. The sample was composed of 485 children and adolescents (398 boys and 87 girls) with a mean age of 6.14 years (SD = 3.46). Among the 485 children 335 mothers and 230 fathers answered the questionnaire about QoL. We examined differences in the sample of 485 included in the analysis with the full baseline cohort of 876. Table 1 gives descriptive statistics for the sample and observations included in the model. Child behavior, parental stress and the impact on QoL all appear to improve over time since diagnosis, but not so for coping, with no evidence of the acquisition of improved problem solving, nor support where there is some evidence of loss of support. Using the child's age, sex, Vineland Daily Living Skills (DLS), ADOS CSS, and best estimate developmental quotient, maternal age (at birth) and family situation, forward stepwise logistic regression identified significant differences in inclusion in the analyzed sample according to DLS and maternal age. None of these variables were significantly related to subsequent missing data in providing parental self‐ratings (stress0) or follow‐up data (CBCL2).

**TABLE 1:** Summary statistics for baseline and second assessment of ELENA cohort.

	Assessment wave					
	Baseline			Wave 2		
	N	Mean	SD	N	Mean	SD
Child age – years	485	6.14	3.46	388	9.22	3.50
CBCL opposition						
Preschool	261	5.00	2.86	30	3.67	2.81
School‐age	224	4.17	2.63	208	3.40	2.64
Mother stress	311	97.16	24.02	178	90.33	25.03
Father stress	223	93.59	23.04	120	88.53	26.06
Mother IoQoL	335	55.91	16.76	172	49.67	17.83
Father IoQoL	230	49.77	16.40	116	46.37	17.48
Mother problem coping (prob0)	306	31.21	4.99	165	30.90	5.40
Mother support coping (supp0)	305	21.57	5.60	171	20.69	5.61
Father problem coping (prob0)	225	29.29	5.47	114	29.04	6.62
Father support coping (supp0)	232	19.84	5.53	113	18.94	5.93

Table 2 shows the correlation among the variables included in the models, the upper triangle being those for mothers and the lower triangle for fathers. For both mothers and fathers the correlations are all generally high, except for the more modest and often small correlations involving problem coping and social support coping. The association of child behavior with QoL is evident for both parents but those with problem solving and social support are much smaller.

**TABLE 2:** Pairwise correlations: mothers lower triangle, fathers upper triangle.

Variables	(1)	(2)	(3)	(4)	(5)	(6)	(7)	(8)
(1) cbcl0		**0.59**	−0.10	−0.09	**0.48**	**0.43**	**0.34**	**0.36**
(2) cbcl2	**0.59**		−0.08	−0.00	**0.35**	**0.48**	0.24	**0.41**
(3) prob0	−0.10	0.00		**0.51**	−0.21	−0.11	−0.16	0.01
(4) supp0	0.05	0.09	**0.37**		−0.06	−0.06	−0.03	0.04
(5) stress0	**0.53**	**0.32**	**−0.29**	−0.06		**0.66**	**0.76**	**0.48**
(6) stress2	**0.49**	**0.55**	−0.25	−0.04	**0.69**		**0.58**	**0.72**
(7) IoQol0	**0.33**	**0.27**	−0.16	−0.02	**0.71**	**0.56**		**0.55**
(8) IoQol2	**0.28**	**0.35**	−0.15	−0.03	**0.49**	**0.74**	**0.62**	

*Note*: Bold *p* < 0.001.

### Model of parental stress mediating the effects of child behavior on impact on Quality of Life

The model of Figure 2 fitted well to the data from mothers (RMSEA = 0.0001, χ^2^(3) = 2.65, *p* = 0.448, CFI = 1.00). Table 3 shows the estimated unstandardized path coefficients in column 1. Child oppositional behavior was strongly related to maternal stress at baseline and follow‐up, and stress strongly related to QoL at baseline and follow‐up, the latter having accounted for any persisting effects of their pre‐existing associations at baseline. The indirect effect of contemporaneous oppositional behavior on the impact on QoL was significant at both baseline (unstandardized mediated effect 4.52*0.51 = 2.31, CI 1.83 to 2.79; standardized 0.38) and follow‐up (unstandardized 3.78*0.57 = 2.15, CI 1.33 to 2.98; standardized 0.32) and in both cases the residual or controlled direct effects in Table 3 were negative. Though the direct effect paths from baseline oppositional behavior to both follow‐up stress and QoL are negative, the model estimated total effects accounting for all indirect paths as significantly positive, consistent with the correlations of Table 2 (unstandardized 3.80, *p* =< 0.001 for stress2 and 1.19, *p* = 0.05 for IoQol2). Adding further covariate adjustment for age and sex to all the equations of the model of Figure 2 made little change to these estimates (data not shown). A table of coefficient estimates from a model with inverse probability participant weights to account for the selection from the full cohort is shown in the Supporting Information (Table S1). They differ little from the unweighted estimates.

**TABLE 3:** Non‐standardized path coefficients, significance and 95% confidence intervals.

		Mothers	Fathers	Mothers	Fathers	Mothers	Fathers
Variable^#^		Model of Figure 2	Model of Figure 2	Model + coping	Model + coping	Model + moderation	Model + moderation
stress0							
cbcl0		4.52***	4.00***	4.30***	3.87***	5.70*	8.39**
		[3.73,5.31]	[3.07,4.92]	[3.53,5.06]	[2.95,4.78]	[0.78,10.62]	[3.15,13.63]
cop0				−1.19***	−0.74**	−1.18***	−0.77**
				[−1.64,−0.74]	[−1.24,−0.24]	[−1.63,−0.72]	[−1.27,−0.28]
cop0 × cbcl0						−0.05	−0.16
						[−0.20,0.11]	[−0.33,0.02]
IoQol0							
stress0		0.51***	0.54***	0.52***	0.54***	0.48***	0.58***
		[0.45,0.57]	[0.47,0.61]	[0.46,0.59]	[0.47,0.61]	[0.32,0.63]	[0.42,0.73]
cbcl0		−0.28	−0.21	−0.30	−0.21	−0.32	−0.20
		[−0.83,0.27]	[−0.78,0.36]	[−0.85,0.24]	[−0.79,0.36]	[−0.88,0.23]	[−0.78,0.38]
cop0				0.17	0.04	0.01	0.16
				[−0.12,0.45]	[−0.23,0.31]	[−0.57,0.59]	[−0.41,0.74]
cop0 × stress0						0.00	−0.00
						[−0.00,0.01]	[−0.01,0.00]
cbcl2							
cbcl0		0.57***	0.57***	0.57***	0.56***	0.38	0.75*
		[0.46,0.67]	[0.46,0.67]	[0.46,0.67]	[0.46,0.67]	[−0.34,1.10]	[0.05,1.45]
cop0				0.02	−0.02	0.01	−0.02
				[−0.05,0.09]	[−0.09,0.05]	[−0.07,0.08]	[−0.09,0.04]
cop0 × cbcl0						0.01	−0.01
						[−0.02,0.03]	[−0.03,0.02]
stress2							
stress0		0.68***	0.67***	0.68***	0.66***	0.68***	0.66***
		[0.55,0.81]	[0.47,0.86]	[0.54,0.82]	[0.47,0.86]	[0.55,0.82]	[0.47,0.86]
cbcl2		3.78***	3.37***	3.80***	3.32***	1.31	2.33
		[2.45,5.11]	[1.46,5.28]	[2.47,5.13]	[1.39,5.24]	[−1.30,3.93]	[−1.16,5.83]
cbcl0		−1.40*	−1.05	−1.42*	−1.00	−1.56*	−1.20
		[−2.75,−0.05]	[−3.01,0.91]	[−2.77,−0.07]	[−2.96,0.97]	[−2.91,−0.22]	[−3.25,0.84]
cop0				0.02	−0.01	0.08	0.08
				[−0.61,0.65]	[−0.65,0.63]	[−0.55,0.71]	[−0.61,0.77]
cop0 × cbcl2						0.09*	0.04
						[0.01,0.17]	[−0.08,0.17]
IoQol2							
stress0	−0.24***		−0.40***	−0.24**	−0.38***	−0.23**	−0.39***
	[−0.38,−0.10]		[−0.59,−0.20]	[−0.38,−0.10]	[−0.58,−0.19]	[−0.37,−0.09]	[−0.59,−0.19]
Ioqol0	0.52***		0.60***	0.51***	0.60***	0.52***	0.61***
	[0.37,0.66]		[0.35,0.85]	[0.37,0.66]	[0.35,0.85]	[0.37,0.66]	[0.36,0.86]
cbcl2	−1.51**		0.73	−1.50**	0.81	−1.57**	0.81
	[−2.61,−0.41]		[−0.45,1.91]	[−2.61,−0.40]	[−0.37,1.98]	[−2.67,−0.47]	[−0.35,1.98]
stress2	0.57***		0.51***	0.57***	0.51***	0.64***	0.47**
	[0.46,0.68]		[0.40,0.63]	[0.46,0.68]	[0.39,0.63]	[0.47,0.80]	[0.18,0.76]
cbcl0	−0.10		−0.22	−0.10	−0.30	−0.02	−0.29
	[−1.04,0.85]		[−1.39,0.96]	[−1.05,0.85]	[−1.47,0.87]	[−0.98,0.93]	[−1.46,0.87]
cop0				−0.01	0.26	0.26	0.10
				[−0.41,0.38]	[−0.12,0.65]	[−0.33,0.85]	[−0.95,1.16]
cop0 × stress2						−0.00	0.00
						[−0.01,0.00]	[−0.01,0.01]
chi2	2.65		1.27	2.62	1.15	NA	NA
df	3.00		3.00	3.00	3.00	NA	NA
p	0.45		0.74	0.45	0.77	NA	NA
N	485.00		485.00	485.00	485.00	485.00	485.00

*Note*: 95% confidence intervals in brackets, *p < 0.05, **p < 0.01, ***p < 0.001, NA not applicable, # Numerical suffix indicates assessment wave of variable.

### Predictions for behavior trajectory scenarios

Most SEM analyses focus their attention on explaining within‐sample covariation, but it is also helpful to consider variation in mean levels. Table 4 shows model predicted values for 5 hypothetical children for maternal stress and impact on QoL. For these 5 children we fix the values of their behavior scores at baseline and follow‐up, one being a reference child with average scores and the other four children with values representing persistent high, declining, increasing and persistent low behavior problems.

**TABLE 4:** Predicted values for mothers of 5 children with hypothetical values of baseline and follow‐up CBCL Oppositional‐Defiant age and sex regression adjusted raw scores.

CBCL history Baseline F‐UP CBCL0 CBCL2	Baseline Mother Stress0	Baseline Mother IoQoL0	Follow‐up Mother Stress2	Follow‐up Mother IoQoL2
Mean Mean 0.21–0.48	98.01	55.85	91.47	50.33
+1SD +1SD	110.37	61.40	106.12	54.33
+1SD–1SD	110.37	61.40	85.99	50.88
−1SD +1SD	85.65	50.30	96.95	49.78
−1SD −1SD	85.65	50.30	76.81	46.33

In the first scenario for a child whose behavior scores equal the mean at each time point, this is associated with a seven‐point reduction in mothers' stress and six‐point reduction in IoQoL score at follow‐up. For a child with behavior scores persistently one standard deviation above these mean values, a mother would be expected to report a 12‐point higher level of stress at baseline and a 15‐point higher level at follow‐up than mothers of mean‐valued children, even though the absolute stress score falls by 4 points. Nonetheless, while her IoQoL rating shows a six‐point elevation at baseline, it falls by seven points at follow‐up. This would suggest that familiarization with behavior problems has allowed adaptation or acceptance that reduces the IoQoL. For a child with a much reducing behavior score (+1SD to –1SD), the baseline stress and IoQoL scores fall substantially over time to below or close to the stress and IoQoL of the parents of the mean child. For a child with worsening behavior, unsurprisingly stress rises by 11 points, substantially above that of a mother of an average child but still well below that of a mother with persisting behavioral problems. The change in IoQoL is modest, the effects of increased stress possibly being compensated by the effects of familiarization. For the behaviorally improving child a reduction in parent stress of nine points goes along with a reduction in four points in IoQoL at follow‐up.

### The effects of coping strategies

Problem coping style may influence stress and impact on QoL directly. As shown in third column of estimates in Table 3, better baseline problem solving was significantly associated with lower baseline stress but not child behavior, nor was any effect on the three follow‐up measures found.

The absence of main effects did not rule out possible moderation of coping strategy on the relationship between child behavior and stress (i.e. the interaction of child behavior and coping style predicting stress or moderated mediation) and might influence the relationship between stress and impact on QoL (i.e. the interaction of stress and coping style predicting impact on QoL). Adding five interactions to test for moderation also gave only one as significant, that of the interaction with wave 2 CBCL predicting stress at wave 2, but in the unexpected direction (oppositional behavior being more stress making for those with better coping). Table S1 displays very similar estimates obtained after weighting back to the original recruited cohort. A two‐group model fitted to high and low coping groups (median split) also indicated nominally significantly different effect of child behavior on stress at wave 2.

Similar analysis examined effects of baseline social support. A two‐group model using social support (median split) was associated with lower baseline maternal stress (*p* < 0.001) but had no additional main effect at follow‐up. An interaction with follow‐up behavior on follow‐up stress (*p* < 0.001) suggested possible moderation suggesting that persisting oppositional behavior for those who had reported good support was especially stressful.

We also extended the mediation model as shown in Figure S1 and Table S2, to consider whether problem solving at baseline and follow‐up might provide an alternative or additional mediational path. At baseline, problem solving was clearly associated with baseline behavior, stress and impact on QoL, but this last was largely indirect through stress. The estimates for the mediational paths from behavior to stress to IoQoL were little changed from the single mediator model of Table 3 (e.g. mother's wave 2 mediated effect estimate 2.16, previously 2.15). Unlike for the stress mediator, at follow‐up no significant direct, indirect or total effects of problem solving on stress nor IoQoL were found. Fitting the same model with social support in place of problem solving gave no significant direct, indirect or total effects on stress or impact on QoL at either time. For both problem coping and social support measures, the significant baseline paths on behavior at baseline were not replicated at follow‐up, raising questions as to their causal influence on behavioral development.

### Results for fathers

The results for fathers were very similar, both in the model fit, effects that were significant, and in their direction of effect. The indirect effect of contemporaneous behavior on QoL was significant at both baseline (unstandardized 4.00*0.54 = 2.16, CI 1.59 to 2.73; standardized 0.32) and at wave 2 (unstandardized 3.37*0.51 = 1.73, CI 0.55 to 2.91; 0.25), and coping significantly associated with lower baseline stress but, unlike where one of the five possible interactions was significant for women, fathers' coping showed none as significant. The extended mediation model of Figure S1 also gave similar results to that of mothers (e.g. effect mediated via stress 1.71, previously 1.73). Better problem solving was associated with less stress at baseline but only marginally with impact on QoL (total effect *p* = 0.069) and no significant effects at follow‐up. Social support showed no significant associations at either time.

## DISCUSSION

Parents of children with ASD experience significant impact of their child's ASD on their QoL. Prior research has generally found that the severity of challenging behaviors were strong predictors of decreased QoL. The current study attempted to add to the existing literature by exploring how parental stress might serve a buffering function in the relationship between oppositional disorders and QoL, and by using longitudinal data to strengthen the evidence for possible causal effects.

First, we found that mean levels of reported child oppositional behavior, parental stress and impact on QoL all improved over the 3 years following diagnosis. While there is a clinical focus on the child at the time of diagnosis, this may also be a particularly challenging time for parents.

Second, we found strong associations between child challenging behaviors, parental stress and parental QoL, congruent with the literature (Anixt et al., 2020; Baghdadli et al., 2014; Matson & Nebel‐Schwalm, 2007; Rattaz et al., 2018; Rutgers et al., 2007). Child oppositional disorders were associated with parental stress, which was associated with parental QoL, for both parents and at both collection time points. At baseline the associations were consistent with stress mediating the relationship between oppositional behaviors in the child and impact on parental QoL. A high level of comorbid disorders in the child would trigger feelings of parental stress, which may then have contributed to the deterioration of parental QoL. As suggested by Chen et al. (2021), high parenting stress may interfere with the ability to respond sensitively and appropriately to children's behavior, thus mediating the relationship between comorbid behaviors and parental QoL. The mediation effects at follow‐up, which control for baseline associations, while somewhat smaller, remained substantial, strengthening the evidence for this mediational effect to be causal.

Third, regarding coping strategies, both better baseline problem solving and social support were significantly associated with lower baseline stress and impact on QoL. However, the multiple mediator models (Table S2) suggested that the primary mediational path was from behavior to stress and very little through either of these coping measures, and the path coefficients at follow‐up also suggested neither measure of coping was associated with improved behavior. Moreover, the tests of interaction (Table 3) showed neither variable moderated the key behavior to stress association at baseline, and at follow‐up, while both were significant, they were in the opposite direction to that expected.

These results somewhat qualify the effectiveness of problem‐focused and social support coping strategies described in the literature (Alostaz et al., 2022; Dabrowska & Pisula, 2010; Gray, 2006; Vernhet et al., 2022). In our study, the effect of challenging behaviors on stress was moderated by baseline social support in mothers (i.e. the interaction of child behavior and coping style predicted stress), but in the unexpected direction. The results suggest that social coping strategies reported as available to mothers do not play a buffering role in the 3 years following ASD diagnosis. One possible explanation might be that persisting challenging behavior 3 years later for those who had reported good coping strategies at diagnosis was especially stressful. It can be assumed that it takes a long time to implement this strategy efficiently and that its mediating or moderating effect may appear later in the child's development. An alternative explanation might be that parents who had high levels of social support at baseline might have not sought out professional interventions to address the problem behavior as quickly because they had social resources to support them. It is also possible that social support initially was high but as the child aged and behavior continued to be a concern, social support decreased and the mothers felt more and more isolated. Indeed, most of the studies in the literature have a cross‐sectional design, studying associations between several variables, for example, association between coping strategies and stress. The present study supports evidence for a causal mediational pathway and shows that parental coping is not sufficient to lead to a decrease in child's challenging behaviors 3 years later.

The mediational findings for fathers were similar to those for mothers, but were somewhat less strong. Neither problem solving nor social support appeared to play any significant role in determining the impact on stress nor QoL. In the literature, social support strategy was found to be more often used by mothers than by fathers (Luque Salas et al., 2017; Rattaz et al., 2023). For example, fathers may use their employment as a way to cope (i.e., going to work to avoid thinking about the problem, which can be related to an ineffective strategy) or seek less support from friends or family when compared to mothers (Grebe et al., 2022).

Unlike many previous analyses, we were able to examine the association between behavior, stress and impact on QoL both in cross‐section and longitudinally, to better understand how the associations reflect causal effects. Nonetheless, we assumed that the direction of effects was from behavior to parental stress and from parental stress to QoL. We did not consider the scope for reverse effects nor of change in coping strategies. In the field of autism, the ELENA cohort is large, but only a subset of parents completed the assessments. Our findings in relation to moderated mediation should be interpreted cautiously in the context of likely limited power. Weighting our analysis to account for selective attrition suggested that our results were robust to this sample selection. The fitted model did not allow for measurement errors. It is usual for mediated effects to increase once account is taken of such errors in the mediator, however shared method variance inducing correlated errors among the measures at each time may have inflated our mediation estimates. As usual results may be subject to bias arising from the presence of confounding variables omitted from the model. Finally, the measures of comorbid disorders, parental stress and coping were collected through parental self‐report, which is a clear limitation. The findings may be stronger if the self‐report measures would have been accompanied by a clinician assessment.

## CONCLUSION

The results of the present study emphasize the crucial role of parental stress in the relationship between the oppositional defiant behavior problems that children with ASD may exhibit and the parents' QoL. The presence of comorbid disorders should be seen as a red flag concerning the risk of a decreased QoL, particularly around the time of diagnosis. While there is strong evidence for the cross‐sectional association of coping strategies with problem behavior and QoL, the evidence for ongoing effects improving outcome is more limited, requiring trial or cohort data. The ELENA cohort is one of the few such data sources for autistic children. Our results provide little evidence of coping strategies having an ongoing causal effect on improving outcomes and though likely not well powered, the only significant moderating effect on the links between children's behaviors and parental stress at wave 2 were negative. One possible explanation would be that social support and problem‐solving strategies of parents may influence initial patterns of child behavior and family adaptation, but once set, do little for subsequent development. In terms of clinical practice, interventions more specifically focused on challenging behavior management, such as functional analysis (Burrell et al., 2020; Gerow et al., 2019), may be more appropriate for parents of children with ASD who also exhibit comorbid externalizing problems. In order to address the parents' needs, interventions focusing on parental involvement and enhancing adaptive parenting would also be of great interest, as it may be one way to improve parental QoL (Musetti et al., 2021). It was shown in the literature that externalizing problems was associated with controlling parenting, which in return thwarts children's needs (Soenens & Vansteenkiste, 2010).

Finally, more research is needed to better understand the role of stress and coping strategies across time, to see if parental stress and coping strategies change as a function of their experience (diagnosis period, childhood, adolescence…). Moreover, in addition to challenging behaviors, it would also be interesting in the future to examine the role of parental stress and coping strategies on anxiety and depression symptoms in youth with ASD. In the future, longitudinal studies will be conducted in the future on the next collection time point of the ELENA cohort, allowing to examine how families adapt to their children's comorbid behaviors over a longer time period.

## AUTHOR CONTRIBUTIONS

**Cécile Rattaz:** Conceptualization; Methodology; Writing ‐ original draft. **Andrew Pickles:** Conceptualization; Formal analysis; Writing ‐ original draft; Writing ‐ review & editing. **Christelle Vernhet:** Conceptualization. **Cécile Michelon:** Data curation; Formal analysis; Methodology. **Marie‐Christine Picot:** Conceptualization; Formal analysis; Methodology. **Amaria Baghdadli:** Conceptualization; Project administration; Supervision; Writing ‐ review & editing.

## CONFLICT OF INTEREST STATEMENT

The authors declare no conflicts of interest.

## TRIAL REGISTRATION

ClinicalTrial.gov, NCT02625116. Registered 04 December 2015, https://clinicaltrials.gov/study/NCT02625116.

## Ethical considerations

The study and informed consent procedure were approved by the Ethics Committee on the Research of Human Subjects at Marseille Mediterranean and the National Commission for Computing and Liberties (CNIL no. DR‐2015‐393). Informed consents were obtained from parents of children to be included in the study.

## Supporting information

Supplementary Material

## Data Availability

The dataset used in this research is not publicly available due to privacy and ethical restrictions.
