# Supplementary material for: Link between children's oppositional behaviors and parental quality of life post‐ASD diagnosis: Mediating role of parental stress and coping strategies
Source: JCPP Adv. 2025 Jan 9;5(3):e12303. doi: 10.1002/jcv2.12303 (PMC12446722; doi:10.1002/jcv2.12303)
Supplement: Supplementary file 1 — Supplementary Material [file JCV2-5-e12303-s001.docx]

**Supporting Information**

**Figure S1.** Extended Mediation Model.


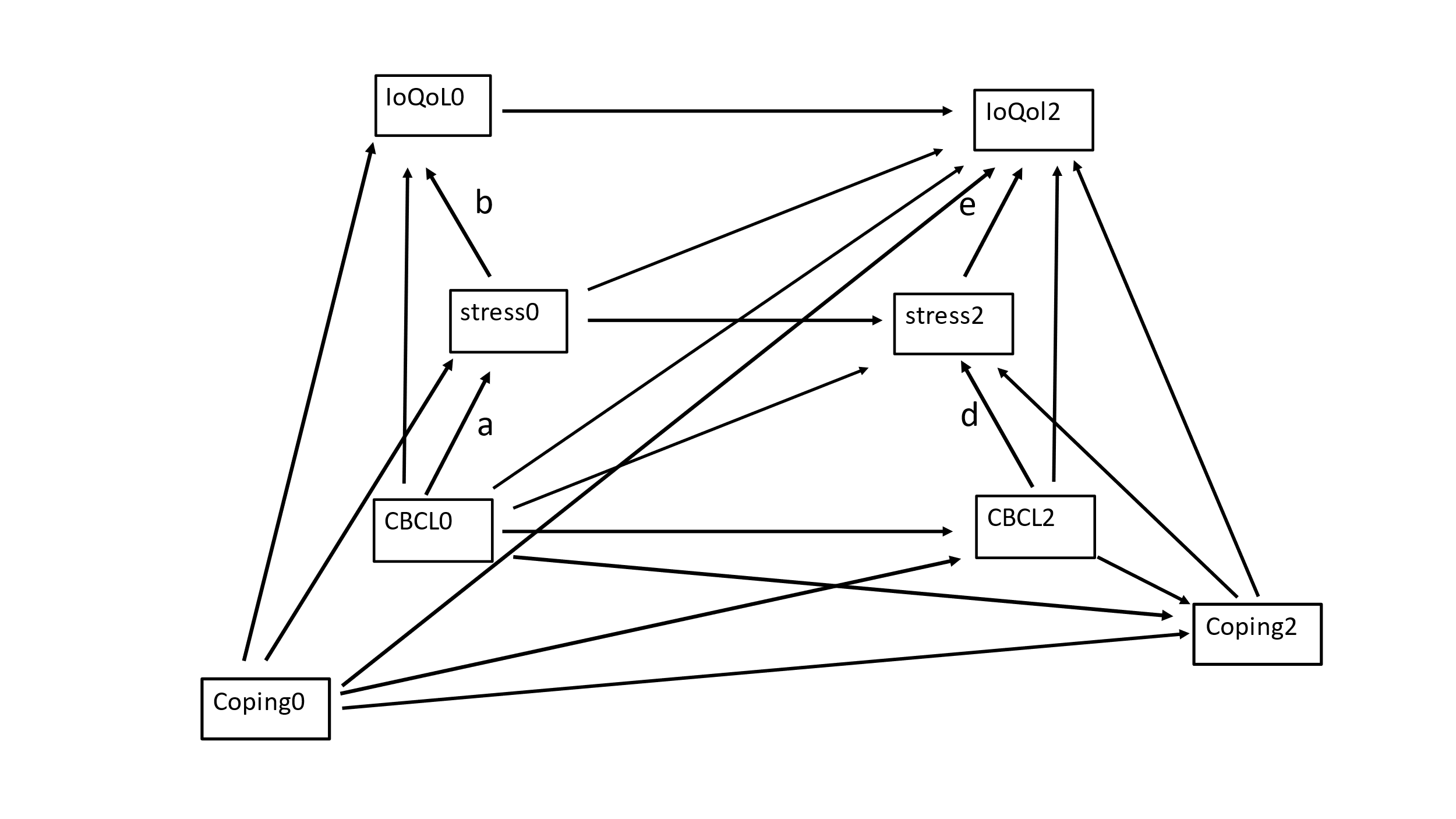


**Table S1.** Estimates from Model of Table 3 weighted for attrition since recruitment.

|  | (1) | (2) | (3) | (4) | (5) | (6) |
| --- | --- | --- | --- | --- | --- | --- |
|  |  |  |  |  |  |  |
| stress0 |  |  |  |  |  |  |
| cbcl0 | 4.40^***^ | 4.06^***^ | 4.27^***^ | 4.00^***^ | 4.23 | 6.50^*^ |
|  | [3.57,5.24] | [3.07,5.06] | [3.46,5.09] | [3.00,5.00] | [-0.71,9.17] | [1.05,11.95] |
| cop0 |  |  | -1.13^***^ | -0.60 | -1.12^***^ | -0.60 |
|  |  |  | [-1.74,-0.51] | [-1.23,0.02] | [-1.74,-0.51] | [-1.24,0.04] |
| cop0 x cbcl0 |  |  |  |  | 0.00 | -0.09 |
|  |  |  |  |  | [-0.16,0.17] | [-0.28,0.11] |
| Ioqol0 |  |  |  |  |  |  |
| stress0 | 0.50^***^ | 0.55^***^ | 0.50^***^ | 0.55^***^ | 0.50^***^ | 0.60^***^ |
|  | [0.43,0.56] | [0.47,0.63] | [0.43,0.57] | [0.47,0.63] | [0.34,0.66] | [0.43,0.77] |
| cbcl0 | -0.34 | -0.18 | -0.35 | -0.19 | -0.35 | -0.16 |
|  | [-1.02,0.34] | [-0.87,0.51] | [-1.02,0.33] | [-0.88,0.50] | [-1.01,0.32] | [-0.84,0.52] |
| cop0 |  |  | 0.04 | -0.02 | 0.03 | 0.17 |
|  |  |  | [-0.27,0.35] | [-0.33,0.29] | [-0.56,0.63] | [-0.45,0.80] |
| cop0 x stress0 |  |  |  |  | 0.00 | -0.00 |
|  |  |  |  |  | [-0.01,0.01] | [-0.01,0.00] |
| cbcl2 |  |  |  |  |  |  |
| cbcl0 | 0.57^***^ | 0.57^***^ | 0.58^***^ | 0.57^***^ | 0.43 | 0.84^**^ |
|  | [0.48,0.67] | [0.48,0.67] | [0.48,0.67] | [0.47,0.67] | [-0.26,1.12] | [0.21,1.47] |
| cop0 |  |  | 0.01 | -0.03 | 0.01 | -0.03 |
|  |  |  | [-0.05,0.08] | [-0.09,0.03] | [-0.07,0.08] | [-0.09,0.03] |
| cop0 x cbcl0 |  |  |  |  | 0.00 | -0.01 |
|  |  |  |  |  | [-0.02,0.03] | [-0.03,0.01] |
| stress2 |  |  |  |  |  |  |
| stress0 | 0.68^***^ | 0.64^***^ | 0.69^***^ | 0.63^***^ | 0.69^***^ | 0.63^***^ |
|  | [0.54,0.82] | [0.39,0.89] | [0.53,0.84] | [0.37,0.89] | [0.53,0.85] | [0.37,0.89] |
| cbcl2 | 3.90^***^ | 3.71^***^ | 3.91^***^ | 3.59^***^ | 1.21 | 3.13^*^ |
|  | [2.61,5.19] | [1.75,5.68] | [2.62,5.20] | [1.66,5.53] | [-2.48,4.90] | [0.16,6.10] |
| cbcl0 | -1.40^*^ | -0.98 | -1.41^*^ | -0.86 | -1.55^*^ | -0.96 |
|  | [-2.59,-0.21] | [-3.21,1.25] | [-2.60,-0.22] | [-3.08,1.36] | [-2.77,-0.33] | [-3.55,1.62] |
| cop0 |  |  | 0.01 | -0.20 | 0.07 | -0.17 |
|  |  |  | [-0.64,0.67] | [-0.94,0.54] | [-0.63,0.77] | [-1.00,0.67] |
| cop0 x cbcl2 |  |  |  |  | 0.10 | 0.02 |
|  |  |  |  |  | [-0.03,0.22] | [-0.13,0.17] |
| qol2 |  |  |  |  |  |  |
| stress0 | -0.23^**^ | -0.37^***^ | -0.23^**^ | -0.35^***^ | -0.22^*^ | -0.34^**^ |
|  | [-0.40,-0.06] | [-0.55,-0.18] | [-0.40,-0.06] | [-0.54,-0.16] | [-0.41,-0.04] | [-0.55,-0.14] |
| qol0 | 0.49^***^ | 0.64^***^ | 0.49^***^ | 0.63^***^ | 0.50^***^ | 0.61^***^ |
|  | [0.36,0.63] | [0.38,0.90] | [0.36,0.63] | [0.38,0.88] | [0.36,0.63] | [0.35,0.88] |
| cbcl2 | -1.53^**^ | 0.83 | -1.53^*^ | 0.93 | -1.58^*^ | 0.94 |
|  | [-2.70,-0.37] | [-0.35,2.00] | [-2.70,-0.36] | [-0.27,2.13] | [-2.86,-0.31] | [-0.25,2.14] |
| stress2 | 0.57^***^ | 0.45^***^ | 0.57^***^ | 0.46^***^ | 0.63^***^ | 0.50^***^ |
|  | [0.44,0.71] | [0.30,0.61] | [0.44,0.71] | [0.32,0.60] | [0.48,0.77] | [0.25,0.74] |
| cbcl0 | -0.01 | -0.03 | -0.01 | -0.15 | 0.05 | -0.15 |
|  | [-0.93,0.91] | [-1.22,1.15] | [-0.93,0.91] | [-1.34,1.04] | [-0.91,1.01] | [-1.33,1.04] |
| cop0 |  |  | 0.02 | 0.38 | 0.24 | 0.53 |
|  |  |  | [-0.30,0.33] | [-0.18,0.94] | [-0.15,0.63] | [-0.56,1.61] |
| cop0 x stress2 |  |  |  |  | -0.00 | -0.00 |
|  |  |  |  |  | [-0.01,0.00] | [-0.01,0.01] |
| chi2_ms | NA | NA | NA | NA | NA | NA |
| df_ms | NA | NA | NA | NA | NA | NA |
| p_ms | NA | NA | NA | NA | NA | NA |
| rmse | NA | NA | NA | NA | NA | NA |
| N | 485.00 | 485.00 | 485.00 | 485.00 | 485.00 | 485.00 |

95% confidence intervals in brackets, ^*^ *p* < 0.05, ^**^ *p* < 0.01, ^***^ *p* < 0.001, , NA not applicable, # Numerical suffix indicates assessment wave of variable.

**Table S2.** Extended Multiple Mediator Model Estimates.

|  | Mother  Problem Solving | Father  Problem Solving | Mother  Social Support | Father  Social Support |
| --- | --- | --- | --- | --- |
|  |  |  |  |  |
| main |  |  |  |  |
| cbcl0 | 4.28^***^ | 3.89^***^ | 4.55^***^ | 4.00^***^ |
|  | [3.52,5.05] | [2.97,4.81] | [3.77,5.34] | [3.07,4.93] |
| mcop0 | -1.20^***^ |  |  |  |
|  | [-1.65,-0.76] |  |  |  |
| fcop0 |  | -0.72^**^ |  |  |
|  |  | [-1.22,-0.22] |  |  |
| msupp0 |  |  | -0.34 |  |
|  |  |  | [-0.75,0.08] |  |
| fsupp0 |  |  |  | -0.06 |
|  |  |  |  | [-0.55,0.42] |
| mqol0 |  |  |  |  |
| mstress0 | 0.52^***^ |  | 0.51^***^ |  |
|  | [0.46,0.59] |  | [0.45,0.58] |  |
| cbcl0 | -0.30 |  | -0.30 |  |
|  | [-0.85,0.25] |  | [-0.85,0.24] |  |
| mcop0 | 0.15 |  |  |  |
|  | [-0.13,0.44] |  |  |  |
| msupp0 |  |  | 0.11 |  |
|  |  |  | [-0.14,0.36] |  |
| cbcl2 |  |  |  |  |
| cbcl0 | 0.57^***^ | 0.56^***^ | 0.57^***^ | 0.56^***^ |
|  | [0.46,0.67] | [0.46,0.67] | [0.46,0.67] | [0.46,0.67] |
| mcop0 | 0.02 |  |  |  |
|  | [-0.05,0.09] |  |  |  |
| fcop0 |  | -0.02 |  |  |
|  |  | [-0.09,0.05] |  |  |
| msupp0 |  |  | 0.03 |  |
|  |  |  | [-0.03,0.10] |  |
| fsupp0 |  |  |  | -0.00 |
|  |  |  |  | [-0.07,0.06] |
| _cons | -1.07 | 0.02 | -1.26 | -0.56 |
|  | [-3.27,1.13] | [-1.94,1.98] | [-2.65,0.13] | [-1.91,0.79] |
| mcop2 |  |  |  |  |
| mstress0 | 0.02 |  |  |  |
|  | [-0.03,0.06] |  |  |  |
| cbcl2 | -0.05 |  |  |  |
|  | [-0.45,0.35] |  |  |  |
| cbcl0 | -0.07 |  |  |  |
|  | [-0.49,0.34] |  |  |  |
| mcop0 | 0.55^***^ |  |  |  |
|  | [0.36,0.73] |  |  |  |
| mstress2 |  |  |  |  |
| mstress0 | 0.69^***^ |  | 0.68^***^ |  |
|  | [0.55,0.82] |  | [0.55,0.82] |  |
| cbcl2 | 3.79^***^ |  | 3.84^***^ |  |
|  | [2.47,5.12] |  | [2.48,5.19] |  |
| mcop2 | -0.18 |  |  |  |
|  | [-0.73,0.38] |  |  |  |
| cbcl0 | -1.43^*^ |  | -1.47^*^ |  |
|  | [-2.78,-0.08] |  | [-2.86,-0.08] |  |
| mcop0 | 0.14 |  |  |  |
|  | [-0.56,0.83] |  |  |  |
| msupp2 |  |  | 0.21 |  |
|  |  |  | [-0.30,0.72] |  |
| msupp0 |  |  | 0.05 |  |
|  |  |  | [-0.55,0.65] |  |
| mqol2 |  |  |  |  |
| mstress0 | -0.24^***^ |  | -0.24^***^ |  |
|  | [-0.39,-0.10] |  | [-0.38,-0.10] |  |
| mqol0 | 0.52^***^ |  | 0.52^***^ |  |
|  | [0.37,0.66] |  | [0.37,0.67] |  |
| cbcl2 | -1.52^**^ |  | -1.52^**^ |  |
|  | [-2.62,-0.41] |  | [-2.62,-0.41] |  |
| mcop2 | 0.07 |  |  |  |
|  | [-0.28,0.43] |  |  |  |
| mstress2 | 0.57^***^ |  | 0.57^***^ |  |
|  | [0.46,0.68] |  | [0.46,0.68] |  |
| cbcl0 | -0.08 |  | -0.07 |  |
|  | [-1.03,0.87] |  | [-1.03,0.89] |  |
| mcop0 | -0.09 |  |  |  |
|  | [-0.53,0.35] |  |  |  |
| msupp2 |  |  | 0.01 |  |
|  |  |  | [-0.32,0.34] |  |
| msupp0 |  |  | 0.01 |  |
|  |  |  | [-0.37,0.39] |  |
| fqol0 |  |  |  |  |
| fstress0 |  | 0.54^***^ |  | 0.54^***^ |
|  |  | [0.47,0.61] |  | [0.47,0.61] |
| cbcl0 |  | -0.21 |  | -0.18 |
|  |  | [-0.78,0.36] |  | [-0.75,0.39] |
| fcop0 |  | 0.04 |  |  |
|  |  | [-0.23,0.31] |  |  |
| fsupp0 |  |  |  | 0.10 |
|  |  |  |  | [-0.16,0.36] |
| fcop2 |  |  |  |  |
| fstress0 |  | -0.03 |  |  |
|  |  | [-0.10,0.03] |  |  |
| cbcl2 |  | -0.11 |  |  |
|  |  | [-0.71,0.49] |  |  |
| cbcl0 |  | 0.18 |  |  |
|  |  | [-0.44,0.80] |  |  |
| fcop0 |  | 0.46^***^ |  |  |
|  |  | [0.26,0.66] |  |  |
| fstress2 |  |  |  |  |
| fstress0 |  | 0.67^***^ |  | 0.67^***^ |
|  |  | [0.47,0.87] |  | [0.47,0.86] |
| cbcl2 |  | 3.29^***^ |  | 3.05^**^ |
|  |  | [1.36,5.23] |  | [1.11,4.99] |
| fcop2 |  | 0.12 |  |  |
|  |  | [-0.51,0.76] |  |  |
| cbcl0 |  | -1.02 |  | -0.91 |
|  |  | [-3.00,0.96] |  | [-2.86,1.05] |
| fcop0 |  | -0.09 |  |  |
|  |  | [-0.80,0.61] |  |  |
| fsupp2 |  |  |  | 0.72 |
|  |  |  |  | [-0.02,1.45] |
| fsupp0 |  |  |  | -0.64 |
|  |  |  |  | [-1.41,0.12] |
| fqol2 |  |  |  |  |
| fstress0 |  | -0.39^***^ |  | -0.42^***^ |
|  |  | [-0.58,-0.19] |  | [-0.61,-0.23] |
| fqol0 |  | 0.62^***^ |  | 0.63^***^ |
|  |  | [0.37,0.86] |  | [0.38,0.88] |
| cbcl2 |  | 0.85 |  | 0.76 |
|  |  | [-0.32,2.01] |  | [-0.41,1.93] |
| fcop2 |  | 0.26 |  |  |
|  |  | [-0.10,0.62] |  |  |
| fstress2 |  | 0.52^***^ |  | 0.52^***^ |
|  |  | [0.40,0.63] |  | [0.40,0.63] |
| cbcl0 |  | -0.37 |  | -0.15 |
|  |  | [-1.53,0.80] |  | [-1.32,1.01] |
| fcop0 |  | 0.15 |  |  |
|  |  | [-0.28,0.57] |  |  |
| fsupp2 |  |  |  | 0.14 |
|  |  |  |  | [-0.30,0.58] |
| fsupp0 |  |  |  | 0.25 |
|  |  |  |  | [-0.23,0.73] |
| msupp2 |  |  |  |  |
| mstress0 |  |  | -0.03 |  |
|  |  |  | [-0.07,0.02] |  |
| cbcl2 |  |  | -0.41 |  |
|  |  |  | [-0.87,0.05] |  |
| cbcl0 |  |  | 0.37 |  |
|  |  |  | [-0.08,0.82] |  |
| msupp0 |  |  | 0.40^***^ |  |
|  |  |  | [0.23,0.57] |  |
| fsupp2 |  |  |  |  |
| fstress0 |  |  |  | 0.00 |
|  |  |  |  | [-0.06,0.06] |
| cbcl2 |  |  |  | 0.34 |
|  |  |  |  | [-0.23,0.91] |
| cbcl0 |  |  |  | -0.24 |
|  |  |  |  | [-0.78,0.31] |
| fsupp0 |  |  |  | 0.53^***^ |
|  |  |  |  | [0.35,0.71] |
| chi2_ms | 2.65 | 1.17 | 3.47 | 3.81 |
| df_ms | 4.00 | 4.00 | 4.00 | 4.00 |
| p_ms | 0.62 | 0.88 | 0.48 | 0.43 |
| rmse |  |  |  |  |
| N | 485.00 | 485.00 | 485.00 | 485.00 |

95% confidence intervals in brackets, ^*^ *p* < 0.05, ^**^ *p* < 0.01, ^***^ *p* < 0.001

**Calculation of Mediated Effects from Estimates of Model in Figure S1 shown in Table S2**

cbcl2 -> stress2 -> qol2

Mother 3.79*0.57=2.16

Father 3.29*0.52=1.71

cbcl2 -> cop2 -> qol2 cbcl2 -> supp2 -> qol2

Mother -0.05*0.07=-0.00 -0.41*0.01=-0.00

Father -0.11*0.26=-0.03 0.34*0.14=0.05

cbcl2 -> cop2-> stress2-> qol2 cbcl2 -> supp2 -> stress2 -> qol2

Mother -0.05*-0.18*0.57=0.01 -0.41*0.21*0.57=-0.05

Father -0.11*0.12*0.52=0.01 0.34*0.72*0.52=0.13

Mediated effects through stress remain substantial, much larger than effects through problem solving (cop2) and through social support (supp2).
